# In Silico Screening of Novel α1-GABA_A_ Receptor PAMs towards Schizophrenia Based on Combined Modeling Studies of Imidazo [1,2-a]-Pyridines

**DOI:** 10.3390/ijms22179645

**Published:** 2021-09-06

**Authors:** Xiaojiao Zheng, Chenchen Wang, Na Zhai, Xiaogang Luo, Genyan Liu, Xiulian Ju

**Affiliations:** 1 Hubei Key Laboratory of Novel Reactor and Green Chemical Technology, School of Chemical Engineering and Pharmacy, Wuhan Institute of Technology, Wuhan 430205, China; zhengxiaojiao916@163.com (X.Z.); Cheryl_CiCi@163.com (C.W.); zhainaaurora@163.com (N.Z.); xgluo@wit.edu.cn (X.L.); 2 School of Materials Science and Engineering, Zhengzhou University, No. 100 Science Avenue, Zhengzhou 450001, China

**Keywords:** GABA_A_ receptor, positive allosteric modulators, 3D-QSAR, docking, pharmacophore, virtual screening

## Abstract

The ionotropic GABA_A_ receptor (GABA_A_R) has been proven to be an important target of atypical antipsychotics. A novel series of imidazo [1,2-a]-pyridine derivatives, as selective positive allosteric modulators (PAMs) of α1-containing GABA_A_Rs with potent antipsychotic activities, have been reported recently. To better clarify the pharmacological essentiality of these PAMs and explore novel antipsychotics hits, three-dimensional quantitative structure–activity relationships (3D-QSAR), molecular docking, pharmacophore modeling, and molecular dynamics (MD) were performed on 33 imidazo [1,2-a]-pyridines. The constructed 3D-QSAR models exhibited good predictive abilities. The dockings results and MD simulations demonstrated that hydrogen bonds, π–π stackings, and hydrophobic interactions play essential roles in the binding of these novel PAMs in the GABA_A_R binding pocket. Four hit compounds (**DS01–04**) were then screened out by the combination of the constructed models and computations, including the pharmacophore model, Topomer Search, molecular dockings, ADME/T predictions, and MD simulations. The compounds **DS03** and **DS04,** with higher docking scores and better predicted activities, were also found to be relatively stable in the binding pocket by MD simulations. These results might provide a significant theoretical direction or information for the rational design and development of novel α1-GABA_A_R PAMs with antipsychotic activities.

## 1. Introduction

Schizophrenia, a persistent and chronic psychiatric disorder, affects about 1% of the worldwide population [1,2]. It is mainly characterized by positive symptoms (delusions, auditory hallucinations, and illusions), negative symptoms (anhedonia, apathy, difficulties with concentration, blunted affect, and social dysfunction), and cognitive abnormalities (impairments in working and verbal memory) [3,4,5]. It is reported that the disease ranked third among the most debilitating diseases in the world [6,7]. The treatment of schizophrenia generally focuses on eliminating the disease-associated symptoms. Presently, the main target of most typical antipsychotics is the subcortical dopamine D2 receptor [8,9,10]. Studies have shown that the long-term use of typical antipsychotics might induce side effects, such as oversedation, anesthesia, obesity, myocarditis, liver damage, hyperprolactinemia, postural hypotension, and extrapyramidal symptoms [11,12]. Hence, there is an urgent need to develop novel antipsychotics, engaging different mechanisms of action from those currently used, which would provide alternatives for schizophrenia patients [13,14].

As early as in 1972, GABAergic dysfunction in schizophrenia was first proposed by Roberts [15]. GABA (γ-aminobutyric acid) was first discovered in mammal brains in 1949 and has been proven to be an important inhibitory neurotransmitter [16]. The ionotropic GABA type A receptor (GABA_A_R) is distributed throughout the central nervous system and mediates most of the rapid inhibitory nerve transmission [17]. The most common GABA_A_R subtype in the human brain consists of two α, two β, and one γ subunits [18]. Moreover, GABA_A_R is a chloride channel receptor, containing multiple binding sites for GABA and a variety of drug ligands, and has been reported to have a significant place in neurophysiology and pharmacology studies [19]. Many previous researches have proved that GABAergic dysfunction was associated with the pathophysiological process of various mental disorders. For instance, postmortem studies indicated lower brain levels of the GABA neurotransmitter in schizophrenia patients [20]. Subsequently, Hofman’s research showed a lower level of mRNA of the α1 subtype of GABA_A_R in the prefrontal cortices of schizophrenia patients [21]. Gonzalo et al. reported that the reduction in GABA/glutamate in the thalamus of individuals at clinical level was a high risk for psychosis [22]. Consequently, the regulation of the GABAergic system, through α1 subunit GABA_A_R (α1-GABA_A_R), may be a potential approach for the treatment of schizophrenia. Furthermore, pharmacological studies have found that zolpidem, as a selective α1-GABA_A_R-positive allosteric modulator (PAM), displayed antipsychotic-like effects in a rat at low dose level, comparable to the second-generation antipsychotic risperidone [23,24,25].

**Figure 1:**
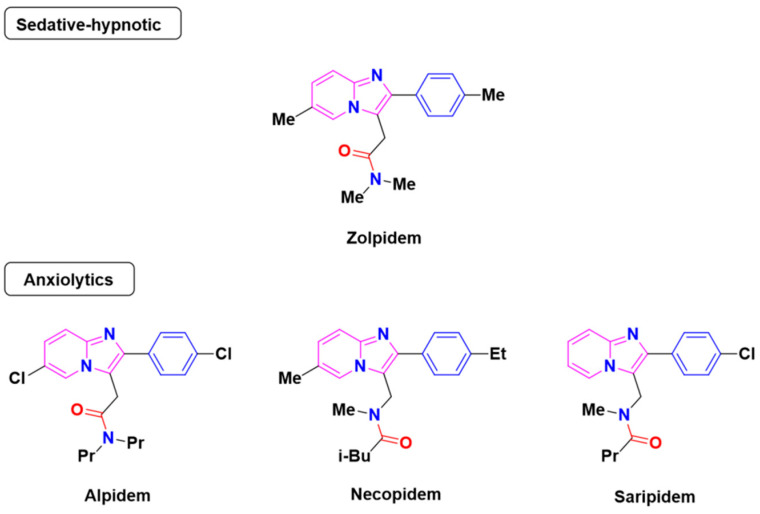
Chemical structures of different α1-GABA_A_R PAMs. Zolpidem and its analogues contain an imidazopyridine (magenta), an amide moiety (red) and an aromatic ring (blue).

Recently, Monike’s group found a series of potential antipsychotics using zolpidem as a point of entry. Subsequently, a series of fluorinated imidazo [1,2-a]-pyridines had been found to be α1-GABA_A_R PAMs with antipsychotic activities [26,27]. Additionally, many structural modifications of the imidazopyridine core have been found [28,29,30], and the most significant α1-GABA_A_R PAMs effect was imidazo [1,2-a]-pyridine compounds with aromatic and amide groups. As shown in Figure 1, zolpidem was an ancient sedative hypnotic pill, and alpidem, necopidem, and saripidem were marketed as anxiolytic drugs successively [31]. Zolpidem and its analogues selectively interact with α1-contaning GABA_A_Rs by the three key pharmacophores, including an imidazopyridine (magenta), amide moiety (red), and aromatic ring (blue).

**Figure 2:**
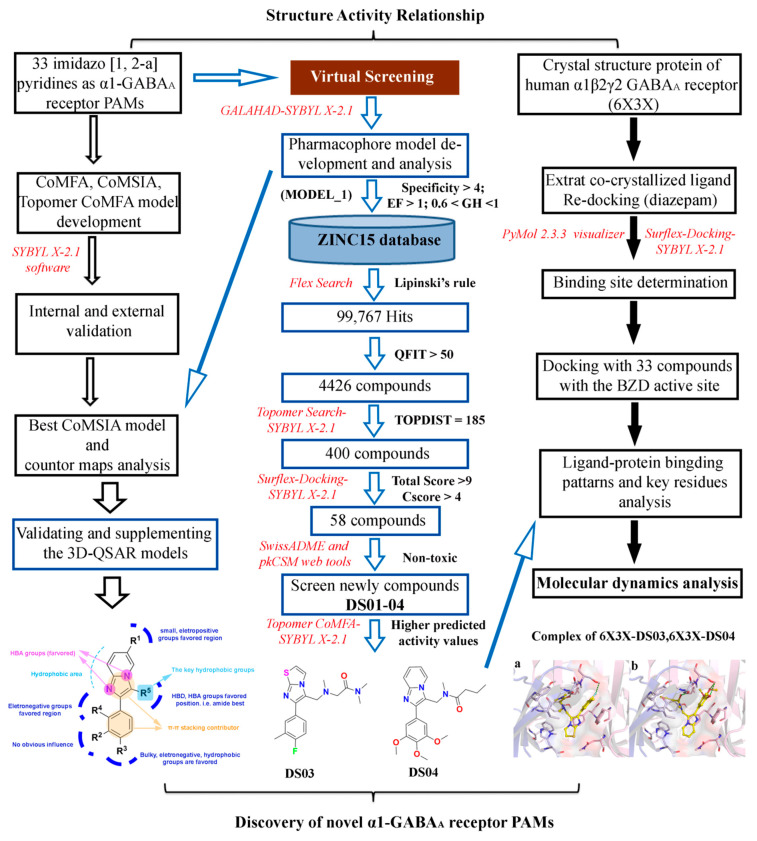
Flow chart of computational drug design for the discovery of novel α1-GABA_A_R PAMs (the used softwares are marked with red italics).

To better explore the crucial pharmacological characteristics of the newly found α1-GABA_A_R PAMs, and design more-efficient antipsychotic agent leads, herein, 33 imidazo [1,2-a]-pyridine PAMs of GABA_A_Rs were selected to perform a systematic modeling study, including three-dimensional quantitative structure–activity relationship (3D-QSAR) models, pharmacophore models, molecular dockings, and molecular dynamics (MD). This study could be clearly illustrated by the following flow chart (Figure 2). To find novel α1-GABA_A_R PAMs, virtual screening was then performed based on the best pharmacophore model combined with Topomer Search, molecular dockings, and ADME/T predictions. MD simulations were subsequently utilized to monitor the real-time dynamic conformation of screened hits and the stabilization of protein–ligand complexes. Our research results might provide important information and more alternatives for the design and exploration of novel GABA_A_R PAMs.

## 2. Results and Discussion

### 2.1. The Statistical Analysis of the 3D-QSAR Models

In the present study, the dataset of 33 imidazo [1,2-a]-pyridines as α1-GABA_A_R PAMs were selected from the published literatures [26,27]. Their chemical structures and activities are given in Table 1. To better understand the QSAR of these novel imidazo [1,2-a]-pyridines as α1-GABA_A_R PAMs, in the present study, the 3D-QSAR models, including comparative molecular field analysis (CoMFA), comparative molecular similarity indices analysis (CoMSIA), and Topomer CoMFA, were established. The statistical parameters of all the models are presented in Table 2 and Table 3, respectively, using the same training (25 molecules) and test (8 molecules) sets. Meanwhile, the Topomer CoMFA model was also employed for the R-group research.

Internal and external validation parameters were important criterions for evaluating the quality and credibility of the 3D-QSAR models. In this study, the cross-validation correlation coefficient (q^2^) value of the CoMFA and Topomer CoMFA models were 0.808 and 0.857, respectively, the non-cross-validated correlation coefficient (R^2^) value were 0.955 and 0.911, respectively, and the optimum number of components (ONC) were 7 and 15, respectively. These internal validation parameters of the two models satisfied the standards. In the CoMFA model, the contribution rates of the steric (S) and electrostatic (E) fields were 37.3% and 62.7%, respectively, suggesting that the contribution of the E field was more crucial. Additionally, the CoMSIA model could be evaluated by any combination of S, E, hydrophobic (H), hydrogen bond donor (HBD), and hydrogen bond acceptor (HBA) fields. Herein, fourteen different CoMSIA models were built and their statistical results are summarized in Table 2. The CoMSIA-SEHAD model that gave relatively reasonable parameters of internal validation (q^2^ = 0.862, R^2^ = 0.980, ONC = 13) was chosen as the optimal model for further analysis. In the CoMSIA-SEHAD model, the contributions of the S, E, H, HBA, and HBD fields were 6.8%, 18.0%, 20.8%, 40.7%, and 14.7%, respectively, suggesting that the E, H, and HBD fields played important roles in this CoMSIA model. It was worth mentioning that the r_pred_^2^ values of the CoMFA, Topomer CoMFA, and CoMSIA models were 0.879, 0.935, and 0.927, respectively, indicating that these three models have reasonable predictabilities. All the validation parameters within the standard ranges in Table 2 and Table 3 suggested that the established 3D-QSAR models were robust and reliable.

**Figure 3:**
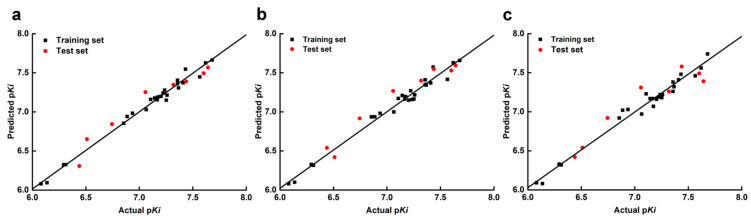
The scatter plots of actual vs. predicted *pKi* values of all used imidazo[1,2-a]-pyridine derivatives based on the CoMFA (**a**), CoMSIA (**b**), and Topomer CoMFA (**c**) models.

The scatter plots of the actual and predicted activities of all the compounds, by the three models, are shown in Figure 3. The statistical points of all the compounds showed a good linear correlation, which further proved that the 3D-QSAR models have high reliability for predicting the activity of those imidazo [1,2-a]-pyridines as GABA_A_R PAMs.

### 2.2. 3D-QSAR Contour Map Analysis

**Figure 4:**
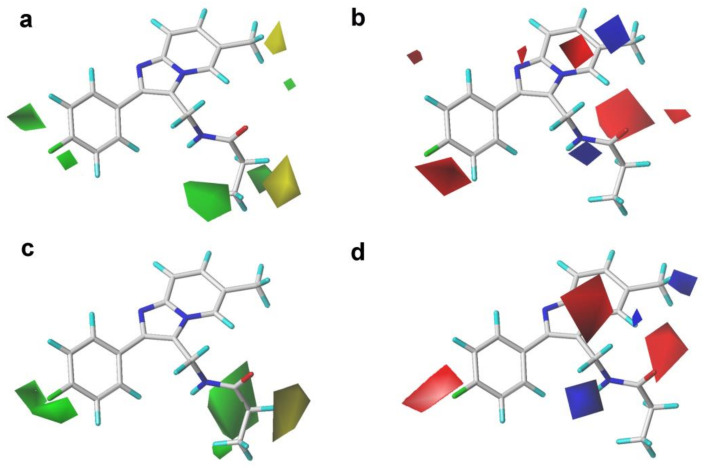
Contour maps of the steric and electrostatic fields of the CoMFA and CoMSIA models with compound **14** as a template. (**a**) The steric contour map of the CoMFA model; (**b**) the electrostatic contour map of the CoMFA model; (**c**) the steric contour map of the CoMSIA model; (**d**) the electrostatic contour map of the CoMSIA model.

**Figure 5:**
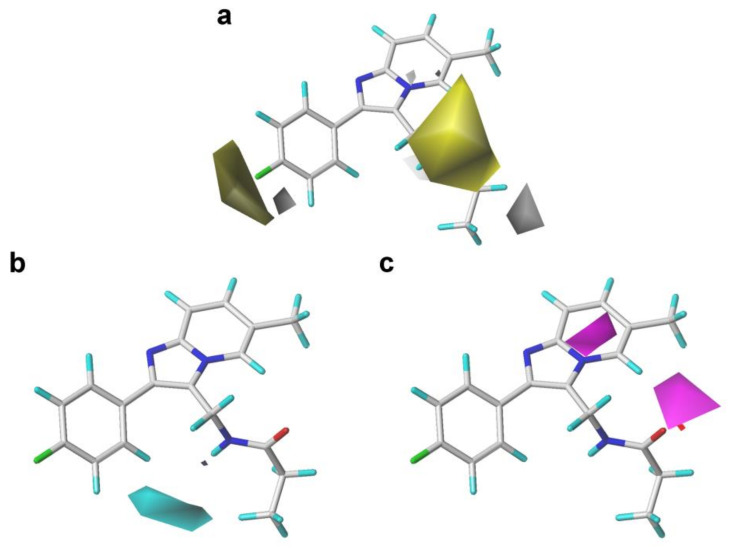
Contour maps of the optimal CoMSIA model with compound **14** as a template. (**a**) The hydrophobic field. (**b**) The hydrogen bond donor field. (**c**) The hydrogen bond acceptor field.

**Figure 6:**
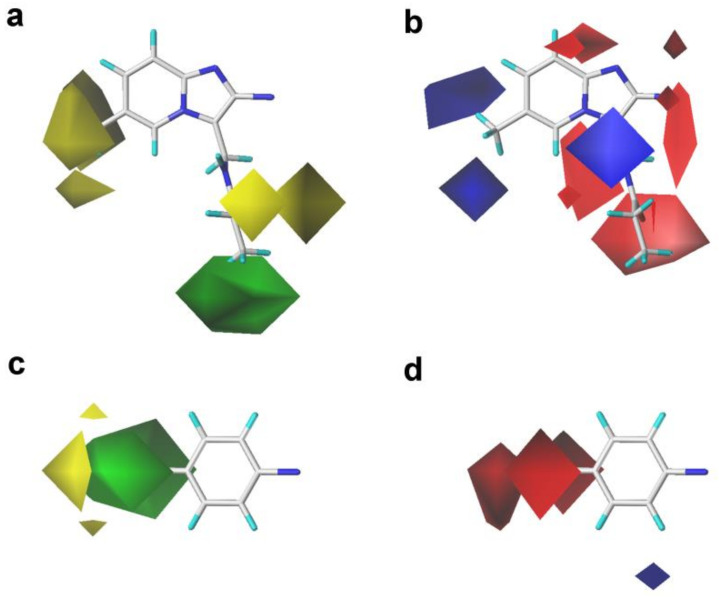
Contour maps of the Topomer CoMFA model with compound **14** as a template. (**a**) and (**c**) Steric fields for part R_a_ and R_b_, respectively; (**b**,**d**) electrostatic fields for part R_a_ and R_b_, respectively.

The CoMFA, CoMSIA, and Topomer CoMFA contour maps with the most potent compound **14** as a reference are shown in Figure 4, Figure 5 and Figure 6, respectively. It can be clearly found that the S and E contour maps of the CoMFA models were similar to those of the CoMSIA model.

As shown in Figure 4, there was a large green contour near the C_4_′ position of the benzene ring, indicating that the introduction of bulky groups at this place might be beneficial for improving the activity. The fact that the methyl or trifluoromethyl substituents at the C_4_′ position were better than the hydrogen atom for the activity could support this result, as illustrated in the following activity orders: **02** (CH_3_) > **03** (H), **17** (CH_3_) > **29** (H), **18** (CH_3_) > **30** (H), and **20** (CH_3_) > **32** (H). A medium-sized yellow block appeared near the C_6_ position of the pyrimidine ring, implying that bulky substituents in this position might not be helpful for the activity, which could be explained as follows: **10** (F) > **07** (CF_3_) and **06** (F) > **09** (CF_3_).

In the S field contours of the CoMSIA model, a large green contour covering the C_3_ position substituent was observed, indicating that bulky volume groups might be more beneficial for the activity at this place. However, there were medium-sized yellow contours around the C_3_ position groups, implying that the bulky groups introduced in this area might be disadvantageous for the bioactivity. The volume of the green color block was slightly larger than that of the yellow contour. In consequence, the influence of the green color block on the S field effect in this area was the primary consideration. A similar situation was also presented in the S field of the CoMFA model. Based on the above analysis of the S field color block, we found that further structural modification using bulky substituents at this position might be favorable for the increment of activity. For instance, the introduction of the 3-methylpropanamide group at this position was more beneficial to the activity than the 3-methylacetamide and 3-methylisobutyramide groups. This could be confirmed by the activity order of the following compounds: **14** (propionamide) > **13** (acetamide) > **15** (isobutyramide), **18** (propionamide) > **17** (acetamide) > **19** (isobutyramide), and **22** (propionamide) > **21** (acetamide) > **23** (isobutyramide).

The E field contours of the CoMFA and CoMSIA models are shown in Figure 4. A medium-sized blue contour was directly opposite to the pyrimidine ring, indicating that the introduction of electronegative substituents at this area might be unfavorable to the activity. This might explain why the activity of compound **01** (CH_3_) was higher than that of compound **07** (CF_3_). It was also found that the introduction of the F atoms at the C_3′_ position of the pyrimidine ring had no significant effect on the activity of those PAMs. A red block was close to the C_2′_ position group, meaning that the electronegative groups at the C_2′_ position might be advantageous for the activity. This could be verified by the following activity orders: **07** (-F) > **08** (-H) and **10** (-F) > **08** (-H). Meanwhile, a lager red contour was also present near the C_4_′ position of the benzene ring, indicating that electronegative substituents at this area might be advantageous for the activity. This could be certified by the fact that the activity of compound **10** (-F) was better than that of compound **03** (-H). A medium-sized red contour in the CoMFA model and a lager red contour in the CoMSIA covered up the imidazo [1,2-a]-pyridine ring, indicating that the occupation of electronegative groups at this region was advantageous for increasing the activity. Moreover, a medium-sized red contour near the carboxide on the propionamide group (-CO-), and a blue contour in both the CoMFA and CoMSIA models could be observed lying in the nitrogen atom of the propionamide group (-NH-). This result confirmed that the propanamide group was the essential group to this series of compounds, which was consistent with the S field results.

The H field contours are exhibited in Figure 5. A larger yellow contour appeared on the side of the substituent at the C_4_′ position of the benzene ring, indicating that hydrophobic groups at this position could be favorable for enhancing the bioactivity. For instance, the hydrogen atom at the C_4_′ position was replaced by the methyl group, causing the activity of compound **03** (p*Ki* = 6.866) to be less than compound **02** (p*Ki* = 7.602). Meanwhile, in the H field contours of the CoMSIA model, a large yellow contour covering the C_3_ position group highlighted the importance of hydrophobic groups in this region. Besides, at the bottom of the C_3_ position substituent, the presence of a small white contour indicated that the hydrophobic groups at this region might decrease the bioactivity. We observed that the volume of the yellow color block was significantly larger than the white color block; therefore, we mainly considered the influence of the yellow color block on the hydrophobic effect in this area. The fact that the 3-methylpropionamide substituent at the C_3_ position was better for the activity than 3-methylacetamide could explain this result, as illustrated in the following activity orders: **14** (propionamide) > **13** (acetamide), **18** (propionamide) > **17** (acetamide), **22** (propionamide) > **21** (acetamide), **26** (propionamide) > **25** (acetamide), and **30** (propionamide) > **29** (acetamide). The hydrophobicity would become slightly stronger with the length of the alkane chain, which might be compatible with the result of the S field that bulky groups be favorable for enhancing the biological activity at the C_3_ position.

The HBD and HBA contours of the CoMSIA model are given in Figure 5. As shown in Figure 6b, a small cyan contour near the C_4_′ position substituent demonstrated that the presence of HBD groups at this position might favor the activity. This could be corroborated by the following active order: **11** (-F) > **04** (-H). Furthermore, a large cyan contour near the C_3_ position substituent of the imidazole ring, and a medium-sized contour were observed near the 3-methylpropionamide substituent on the imidazole ring, demonstrating that HBA groups were favorable for the activity at these positions. These results could well explain why the activities of compounds **14–33** (-CO-) were superior to those of compounds **07–09** (-NH-). In addition, two medium-sized magenta contours near the N atoms of the imidazo [1,2-a]-pyridine ring manifested that the existence of a HBA group on the common scaffold might be significant for the activity. These observations were consistent with a previous study that the 3-methylamide group and the imidazo [1,2-a]-pyridine ring of those PAMs were essential for the bioactivity, as they could serve as HBDs or HBAs to interact with the GABA_A_R protein [26,27,30].

The contour maps of the S and E fields of Topomer CoMFA (Figure 6) were basically coincident with that of the CoMFA and CoMSIA models, further verifying the reliability of 3D-QSAR models. 

According to the analyses of the 3D-QSAR models, the suitable substituents for increasing the activity of these PAMs at specific regions might be concluded as follows: (1) small group and/or electropositivity at the C_6_ position of the imidazo [1,2-a]-pyridine ring; (2) bulky, negative-charged, and/or hydrophobic groups at the C_4_′ position of the phenyl ring; (3) negatively charged groups at the C_2_′ position of the phenyl ring; (4) hydrophilic groups at the C_3_ position of the imidazo [1,2-a]-pyridine core; (5) 3-methylamide group at the C_3_ position of the imidazo [1,2-a]-pyridine core as HBDs or HBAs; (6) HBDs groups at the *para*-position of the phenyl ring at the C_4_′ position. Generally, the established 3D-QSAR models were dependable, and could provide a theoretical basis for the design and synthesis of novel efficient PAMs.

### 2.3. Molecular Docking

**Figure 7:**
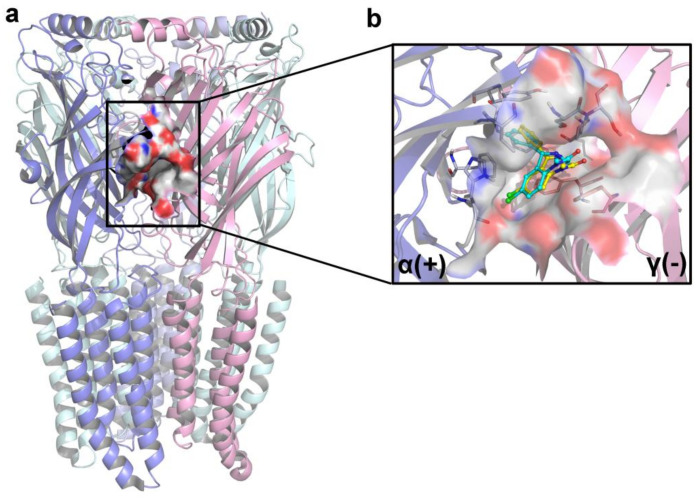
(**a**). The BZD binding pocket of human α1β2γ2 GABA_A_R (PDB ID: 6X3X); (**b**) binding conformations of the original ligand DZP (cyan) and the re-docked DZP (yellow) at the binding site.

A newly crystal structure of the human GABA_A_R α1β2γ2 subtype, in a complex with GABA plus classical benzodiazepines (BZD) diazepam (DZP), has been successfully resolved (PDB ID: 6X3X) in 2020 [32]. In this study, the generated BZD binding pocket of this complex, by the Surflex-Dock method, is depicted in Figure 7a. In order to validate the reliability of the docking method and explore the preferred binding patterns of the test molecules, the co-crystallized DZP was extracted and then re-docked into the BZD binding pocket of the α(+)/γ(−) interface of the human α1β2γ2 GABA_A_R. As depicted in Figure 7b, the conformation of the re-docked DZP almost overlapped with that of the co-crystallized DZP, and their rotational tendencies were basically the same. The root mean square deviation (RMSD) value of the two conformations was 0.43 Å, which was less than 2.0 Å, illustrating that the binding mode was well-founded and could be applied for further study. The two benzene rings of the DZP molecule were found to form π–π stacking interactions with Tyr210 (loop C of α1 subunit) and Phe77 (loop D of γ2 subunit), respectively. Two benzene rings and the Cl substituent were also involved in hydrophobic interactions with surrounding amino acid residues (Phe100, Tyr160, Val203, Tyr210 in α1, and Tyr58, Phe77 in γ2), which might be one of the main reasons for the stable binding of DZP in the GABA_A_R protein. These interactions, such as π–π stacking and hydrophobic interactions between DZP and the 6X3X protein, were in accordance with a previous report [33]. The above-mentioned findings further demonstrated that the docking method was accurate and could be used for the following investigation.

**Figure 8:**
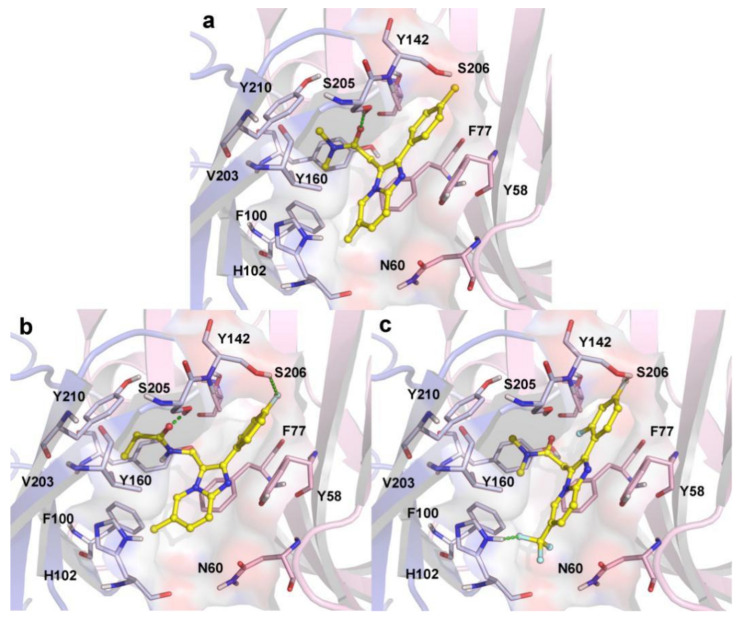
The docking results of zolpidem (**a**), compound **14** (**b**), and compound **8** (**c**) in the binding site of human α1β2γ2 GABA_A_R (PDB ID: 6X3X).

To comprehensively survey the binding mechanism of these novel PAMs, 33 imidazo [1,2-a]-pyridine derivatives (Table 1) were then docked into the GABA_A_R BZD site, using the same method. In general, we found that the docking scores of these PAMs were basically consistent with their activities. The structural models of zolpidem binding at the α(+)/γ(−) interface of 6X3X are displayed in Figure 8a.

Zolpidem was located at the α1/γ2 interface in an oblique insertion, the imidazopyridine ring pointed down in the binding pocket towards His102 (loop A of α1 subunit), and the carbonyl group pointed to loop C. There was a key hydrogen bond formed between the carbonyl oxygen atom and Ser205 in loop C (Ser205-O-H…O-C, 1.8 Å). The dimethyl amide side chain embedded into the hydrophobic pocket that was generated by the surrounding amino acid residues, Phe100, His102, Tyr160, Val203, and Tyr210. Additionally, the π–π stacking interaction was also found between the imidazole ring of zolpidem and the benzene ring of the aromatic residues Phe77 (face-to-face) in loop D. All the observed interactions were consistent with the findings reported by Tikhonova et al. [30,34,35]. Compound **14,** with the highest potency, and compound **08,** with the lowest activity, were selected to concretely analyze the interaction patterns of these PAMs in 6X3X (Figure 8b,c). The docking score of compound **14** (6.91) was higher than that of compound **08** (5.11), which was consistent with their experimental activities. Furthermore, it was noted that compounds zolpidem, **14**, and **08** have similar binding orientations and interactions in the binding pocket. Similarly to zolpidem, the imidazole ring, as the common skeleton of compounds **14** and **08,** formed a π–π stacking interaction with the aromatic Phe77 (γ1). The dimethylamide side chain of compound **08,** and the propanamide moiety of compound **14** were involved in hydrophobic interactions with surrounding amino acid residues. The carbonyl group of compound **14** (Figure 8b) formed a hydrogen bond with the key residue Ser205 (Ser205-O-H…O-C, 1.5 Å). However, this crucial hydrogen bond was not observed in the docking results of compound **08** (Figure 8c). This result was in agreement with the HBA contours of the 3D-QSAR analysis. In addition, the F atom was present at the *para*-position of the phenyl ring of compound **14,** as a HBA formed a hydrogen bond with Ser206 (Ser206-O-H…F-Phenyl, 2.6 Å), and this additional weak hydrogen bonding was also presented in the docking result of compound **08**. The higher docking score of compound **14** might be due to the stronger hydrogen bond formed with Ser205 (α1), and the deeper binding and rotation of the 3-methylpropionamide part in the hydrophobic pocket.

In comparison with compound **14**, compound **08** was observed to lose partial interactions with Ser205 (α1), which might be considered as the main reason that caused its decreased potency. These findings provide evidence to support the essential role of those key residues for the antipsychotic activity in the BZD active site. These results were consistent with the 3D-QSAR analysis, further validating and supplementing the 3D-QSAR models.

### 2.4. Pharmacophore Model

**Figure 9:**
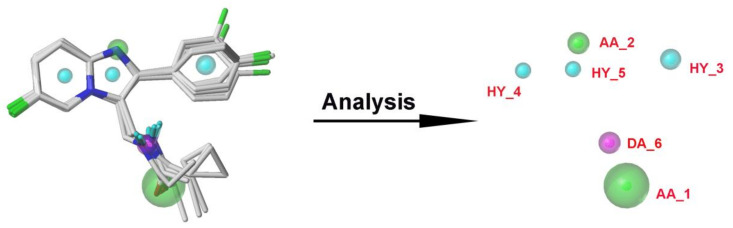
The pharmacophore model constructed with GALAHAD. Green, magenta, cyan, and blue spheres represent hydrogen bond acceptor atoms (AAs), hydrogen bond donor atoms (DAs), and hydrophobes (HYs), respectively.

**Figure 10:**
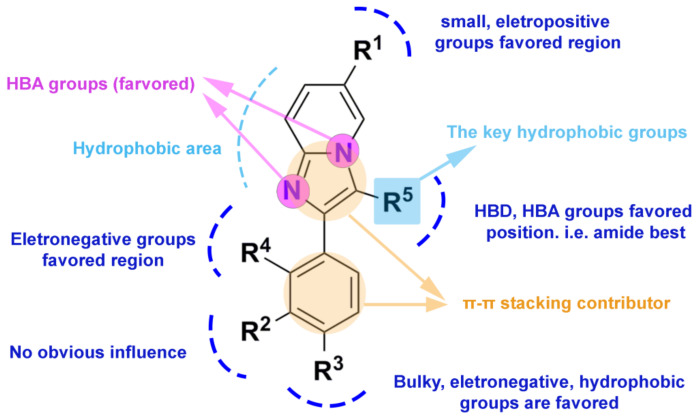
The SARs of imidazo [1,2-a]-pyridine derivatives as GABA_A_R PAMs based on the 3D-QSAR models, molecular docking results, and the best pharmacophore model.

To investigate the key pharmacophore features of these PAMs, in this study, twenty pharmacophore models were generated by aligning and comparing the common features from a test set of ten PAMs, and their statistical results are listed in Table 4. The MODEL_1 was considered to be the optimal pharmacophore model, as it gave the best parameters, including SPECIFICITY = 5.015 (>4), N_HITS = 10, FEATS = 6, PARETO = 0, and ENERGY = 25.5. The decoy set method was then used to verify the quality of MODEL_1, and the results are shown in Table S1. These calculated parameters for MODEL_1 could be concluded as follows: GH = 0.734 (0.6 < GH < 1) and EF = 117.398 (>1). These also demonstrated that the MODEL_1 has powerful ability in discriminating the active compounds from the inactive compounds [36]. Therefore, MODEL_1 was selected to analyze the pharmacological features, and was applied for the following virtual screening. Figure 9 displayed six pharmacophore features using the most potent compound **14** as a reference, including three hydrophobic centers (HYs), two hydrogen bond acceptor atoms (AAs), and one hydrogen bond donor atom (DA). The three HYs were located at the center of the imidazole ring, the pyrimidine ring, and the benzene ring, respectively, suggesting the significance of hydrophobic interactions. The common scaffolds of zolpidem analogues were reported to be indispensable to maintain the activity, which was in agreement with the pharmacophore results that three HYs were distributed in the center of the three aromatic rings. Furthermore, we could observe that the HDs were present on the nitrogen atoms of the propionamide moiety, one HA presented on the oxygen atom of the propionamide side chain, and another HA presented on the nitrogen atom of the imidazole ring of the skeleton. These indicated that the propionamide group structure and the imidazole ring might be important for pharmacological action. To sum up, these findings were in accordance with the 3D-QSAR and docking results, further demonstrating the reliability of this pharmacophore model. A graphical SAR summary of these imidazo [1,2-a]-pyridine derivatives, based on the results of the 3D-QSAR models, the molecular dockings, and the optimal pharmacophore model, is shown in Figure 10.

### 2.5. Virtual Screening Analysis

**Figure 11:**
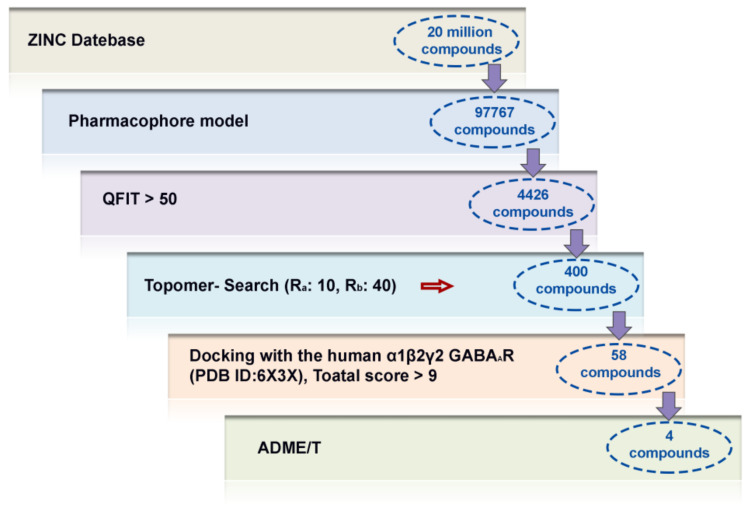
The virtual screening workflow for searching potential antipsychotic agents.

To find potential antipsychotic agent leads, multiple-round virtual screenings were implemented, using the established 3D-QSAR and pharmacophore models in combination with molecular dockings, and the screening workflow is shown in Figure 11. Firstly, the optimal pharmacophore model (MODEL_1) was converted into a UNITY query to screen against the ZINC purchasable database. There were 97,767 compounds that fitted the pharmacophore model, and 4426 compounds with QFIT > 50 were selected for the next round of screening using Topomer Search. In the present work, Topomer Search was employed to screen R groups in the obtained 4426 hit compounds. The screening results were assessed by the TOPDIST and the contribution values of the R groups (TOPCOMFA_R), respectively. Subsequently, 495 R_a_ and 491 R_b_ fragments were gained by Topomer Search, and target fragments with higher contributions were then collected from all the screened R groups using compound **14** as a template. Consequently, 10 R_a_ and 40 R_b_ groups, with the rational TOPDIST and TOPCOMFA_R values, were extracted, and 400 possible compounds were obtained using the rule of permutation and combination. Next, these 400 molecules were docked into the GABA_A_R BZD site, and 58 compounds (docking score > 9 and Cscore > 4) were obtained. To guarantee that newly designed molecules are the feasible drugs, the ADME/T properties of the 58 newly screened compounds were then predicted by the *pkCSM-pharmacokinetics* web tool [37], using zolpidem and compound **14** as reference molecules. Their drug-likeness properties were further predicted using the *SwissADME* online tool [38]. A portion of data was summarized in Table 5. Finally, four newly screened hits (**DS01–DS04**) were obtained through the following parameter criteria: 150 < MW < 500 g/mol; 20 < TPSA < 130 Å^2^; high GI absorption; existent blood–brain barrier (BBB) permeability; existent central nervous system (CNS) permeability (log PS > −2); Caco_2_ permeability > 0.9; intestinal absorption (human) > 90%; lower inhibitory characteristics with CYP450; a low value of total clearance; synthetic accessibility score < 4; respect all drug-likeness rules; and no skin sensitization.

**Figure 12:**
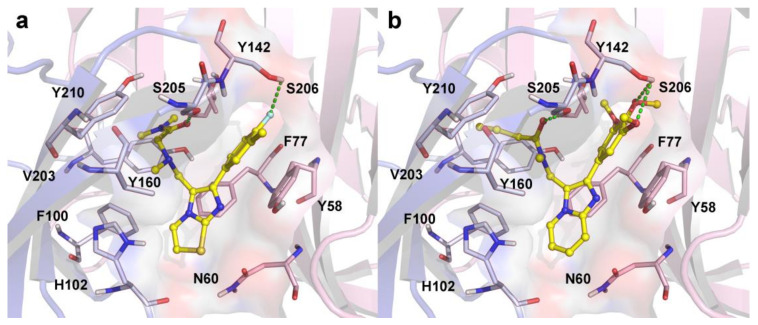
The docking results of the newly screened compounds **DS03** (**a**) and **DS04** (**b**) in the binding site of human α1β2γ2 GABA_A_R (PDB ID: 6X3X).

Table 6 summarizes the chemical structures of four hit compounds (**DS01–DS04**) with their docking scores and predicted p*Ki* values, by the Topomer CoMFA model. The compounds **DS03** and **DS04** were selected for further study, as their predicted activities were higher than those of the other hit compounds and compound **14**. The docking results of compounds **DS03** and **DS04** in the BZD active site of human α1β2γ2 GABA_A_R are depicted in Figure 12. It could be observed that the docking conformations of the compounds **DS03** and **DS04** were basically similar to that of compound **14.**

The important residues within 5 Å of the ligand involved in the interactions are Phe100 (α1), His102 (α1), Tyr160 (α1), Val203 (α1), Ser205 (α1), Ser206 (α1), Tyr210 (α1), Tyr58 (γ2), and Phe77 (γ2). Similarly to zolpidem and compound **14**, the carbonyl group in the side chain of compound **DS03** formed a hydrogen bond with Ser205 in loop C. Another hydrogen bond was found between the F atom in the *para*-position of the benzene ring and Ser206. As for compound **DS04** (Figure 12b), two hydrogen bonds with residues Ser205 and Ser206 in α1 subunit were also found. Meanwhile, two hit compounds deflected closer to critical residues (e.g., Phe100, His102 in loop A; Tyr160 in loop B; Val203, Tyr210 in loop C) and had multi-hydrophobic interactions with them. In addition, π–π stacking was also generated between the imidazole rings of both compounds and the phenyl groups of the aromatic residue Phe77 (face-to-face) in loop D. A similar π–π stacking interaction was also found between the benzene ring and Tyr58. Therefore, it could be predicted that the hit compounds might have a stronger binding affinity to the 6X3X protein than compound **14**. The above-mentioned results revealed that these compounds ought to act as a potential scaffold for designing novel α1-GABA_A_R PAMs, and might provide meritorious reference for the rational designing of novel antipsychotics leads.

### 2.6. Molecular Dynamics Simulation

In order to further clarify the binding interactions between the screened ligands and the GABA_A_R protein, the best docking conformations of zolpidem, **14**, **DS03**, and **DS04,** in the complex with the 6X3X protein, were subjected to 30 ns MD simulations, respectively.

**Figure 13:**
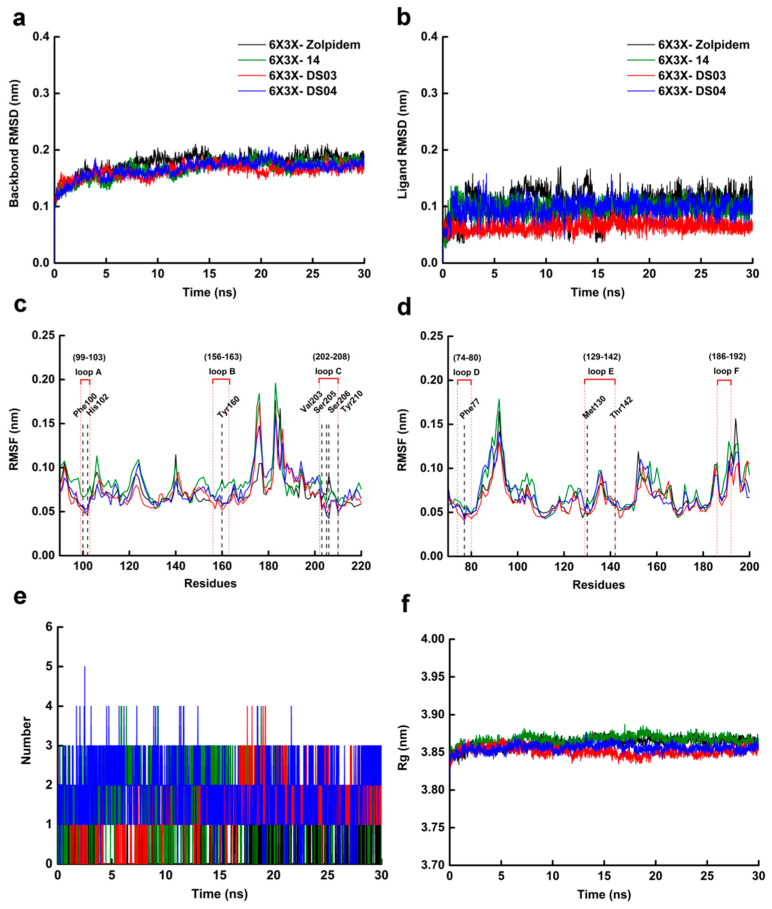
The results of the 30 ns MD simulations of four protein–ligand complexes 6X3X-zolpidem (black), 6X3X-**14** (green), 6X3X-**DS03** (red) and 6X3X-**DS04** (blue). (**a**) The RMSDs of the 6X3X backbone atoms. (**b**) The RMSDs of compounds (zolpidem, compounds **14, DS03** and **DS04**). (**c**) The residue RMSFs of loop A–loop C in the 6X3X. (**d**) The residue RMSFs of loop D–loop E in the 6X3X. (**e**) The numbers of hydrogen bonds formed between the 6X3X and compounds (zolpidem, compounds **14, DS03** and **DS04**). (**f**) Radius of Rg values of backbone atoms.

The time-dependent behavior of the complexes 6X3X-**DS03** and 6X3X-**DS04** in the 30 ns simulation trajectory frames were analyzed using the complexes 6X3X-zolpidem and 6X3X-**14** as the comparisons, and their results were summarized in Figure 13. The RMSD analysis usually provides key information about the convergence and stability of these complex systems [37]. We could observe the RMSD values of the protein backbone atoms of four complexes stabilized after 5 ns MD simulations, converging at 0.20 nm (Figure 13a). From the thorough analysis of the trajectory frames, the RMSD values of the backbone atoms of complexes 6X3X-**DS03** and 6X3X-**DS04** were slightly lower than those of the other complexes. For the ligand RMSD values (Figure 13b), they fluctuated greatly in the 6X3X-zolpidem complex at the beginning of 20 ns, whereas they fluctuated at about 0.06 nm and 0.09 nm in the complexes 6X3X-**DS03** and 6X3X-**DS04**, respectively. Similarly, the RMSD values of four ligands could reach equilibrium finally. Compared to zolpidem and compound **14**, compounds **DS03** and **DS04** had relativity lower RMSD values and fluctuation, indicating a stable interaction or binding to the 6X3X protein. Moreover, the root mean square fluctuation (RMSF) of the Cα residues was computed to identify the structural changes induced by the ligand binding. The RMSF values of the residues in the loops A–F, where the BZD binding site is located, are depicted in Figure 13. From the RMSF trajectories, it is evident that the RMSF values of the most residues in the four complex systems showed similar fluctuations, which demonstrated that four compounds had the analogical binding modes. The RMSF values of the key residues of four complex systems are summarized in Table S2. The critical residues Phe100 (loop A), His102 (loop A), Tyr160 (loop B), Val203 (loop C), Ser205 (loop C), Ser206 (loop C), Tyr210 (loop C), and Phe77 (loop D), in the binding pocket, had relatively low RMSF values (<0.08). These results revealed that the critical residues in the four systems exhibited low flexibility, illustrating that the key interactions of all the compounds in the binding pocket might maintain stability.

Figure 13e shows that the hydrogen bond numbers between the ligand and protein in the complexes 6X3X-zolpidem, 6X3X-**14**, 6X3X-**DS03**, and 6X3X-**DS04,** during the MD simulation, fluctuated 0–1, 0–3, 0–4, and 0–4, respectively. It is worth noting that the hydrogen bond numbers of the complexes 6X3X-**DS03** and 6X3X-**DS04** were slightly higher than that of the complexes 6X3X-**14** and 6X3X-zolpidem. Furthermore, the hydrogen bond numbers in the complexes 6X3X-zolpidem, 6X3X-**14**, 6X3X-**DS03,** and 6X3X-**DS04** maintained in 1, 1, 2, and 3 after 10 ns, respectively. This indicated that the hit compounds **DS03** and **DS04** might be more stable than zolpidem and compound **14** in the binding pocket. Afterwards, we estimated the gyration radius (Rg) of the Cα atoms of the four complex systems, to evaluate the compactness of the protein structure during MD simulations (Figure 13f). All the complexes showed slight fluctuations in the first 20 ns, and the Rg values remained around 3.85 nm finally, suggesting that the protein conformations might be basically stable in the 30 ns dynamic simulation, which was consistent with the RMSD results.

The molecular mechanics-Poisson Boltzmann surface area (MM-PBSA) approach was considered to be one of the most suitable procedures to calculate the binding free energies (ΔG_binding_), which has high accuracy and high computational potency in calculating the binding affinities of ligands with their targets [39]. As shown in Table 7, the ΔG_binding_ values of the compounds zolpidem, **14**, **DS03**, and **DS04** in the GABA_A_R protein were −95.181, −120.055, −131.507, and −126.376 kJ/mol, respectively, which was consistent with the docking results. The van der Waals energy was observed to be the largest contributor to ligand binding, whereas the polar solvation energy was disadvantageous for the binding. In conclusion, zolpidem, compound **14**, **DS03**, and **DS04** could be stable in binding to the 6X3X protein during the whole MD simulation, and the screened hits **DS03** and **DS04** might have better binding stability than zolpidem and compound **14**.

## 3. Materials and Methods

### 3.1. Molecular Construction and Structure Optimization

All simulations and calculations were performed using SYBYL-X 2.1 software (Tripos Inc., St. Louis, MO, USA) running on Windows 10 workstations. The three-dimensional structures of these PAMs were drawn using the SKETCH module and were then optimized with the Tripos force field and Gasteiger–Hückel charges. The *Ki* values of all molecules were converted into p*Ki* by the following formula: p*Ki* = −log*Ki*. The minimization parameters were set with an energy gradient of 0.005 kcal/(mol·Å) and a maximum iteration of 1000 by the Powell method, and the other parameters were set as defaults [40].

### 3.2. 3D-QSAR Model Generation and Alignment

**Figure 14:**
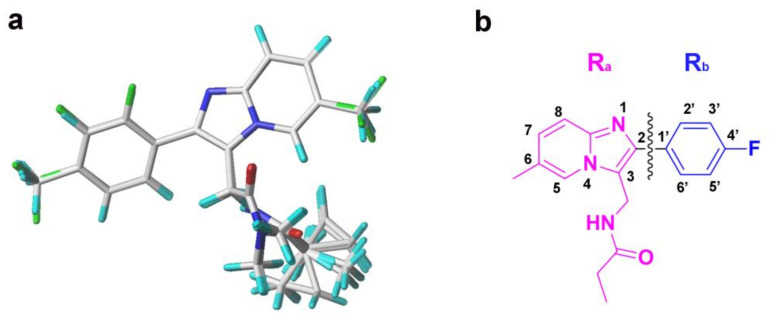
The molecular alignment and fragment for the 3D-QSAR models. (**a**) The alignment of all training set compounds using compound **14** as a template for the CoMFA and CoMSIA models. (**b**) The cutting style of compound **14** for the Topomer CoMFA model; R_a_ group and R_b_ group are colored in magenta and blue, respectively, and the cut position is represented by a wavy line.

The CoMFA and CoMSIA methods were applied to construct 3D-QSAR models. The CoMFA model incorporates two different descriptor fields including S and E fields. In addition to the S and E fields, the CoMSIA model affords H, HBD, and HBA fields [41]. The molecular alignment was the key step for 3D-QSAR models, which impacted the predictability and robustness of the models [42]. In this study, 33 imidazo [1,2-a]-pyridines used for the 3D-QSAR models were randomly divided into a training set of 25 molecules for the model generation and a test set of 8 molecules for the model validation. Compound **14** with the highest potency was utilized as a template for the structure-based alignment (Figure 14a). Topomer CoMFA was a fragment-based 3D-QSAR method that integrates the initial “topomer” methodology and the CoMFA technology [43]. Unlike CoMFA and CoMSIA, the most critical step was to select the split mode or identify the R group in Topomer CoMFA modeling. The prediction accuracy of the Topomer CoMFA model and the reliability of the contour map depend on this step [44]. Each molecule can be divided into several small fragments by cutting the twistable chemical bond, and then the S and E fields of the corresponding fragments could be calculated, respectively. In this study, the Topomer CoMFA model was constructed by using the same training and test sets of the CoMFA model. The Topomer CoMFA model was constructed by dividing compound **14** into two segments, R_a_ (magenta) and R_b_ (blue), as shown in Figure 14. The segmentation position was shown as a black curve in Figure 14b, and the other training set compounds were automatically identified and cut in the same pattern. Subsequently, three-dimensional conformations of the segments were obtained and used for predicting their biological activities or properties.

### 3.3. Analysis and Validation of the QSAR Model

In order to evaluate the reliability and predictive ability of the established 3D-QSAR model, internal and external verification parameters were subsequently statistically analyzed [36]. The q^2^ value and the ONC were obtained by means of leave-one-out cross-validation analysis. The R^2^, the Fisher’s statistic values (F), and the standard error of estimate (SEE), were calculated to assess the predictive ability of models. The above-mentioned parameters were usually considered as internal validation parameters. A model, which is equipped with the following internal parameter ranges: q^2^ > 0.5, R^2^ > 0.6, and SEE << 1, could be considered as a trustworthy model and might have good internal prediction capabilities [45]. Furthermore, external verification parameters, including r_0_^2^, r_0_′^2^, k, k′, r_m_^2^, r_m_′^2^, r_pred_^2^, ∆r_m_^2^, $\bar{r_{m}{}^{2}}$ and RMSE, were further taken into consideration [41]. The QSAR model would be deemed to have excellent external predictive ability only if those statistical parameters met following requirements: r_pred_^2^ > 0.6, r_0_^2^ (r^2^ − r_0_′^2^)/r^2^ < 0.1 or r_0_^2^ (r^2^ − r_0_^2^)/r^2^ < 0.1, 0.85 < k < 1.15 or 0.85 < k′ < 1.15, ∆r_m_^2^ < 0.2, $\bar{r_{m}{}^{2}}$, and > 0.5 [36,45].

### 3.4. Molecular Docking

In this study, the molecular docking was performed using the SYBYL-X 2.1 software and was visualized using PyMol 2.3.3 software (DeLano Scientific LLC, San Carlos, CA, USA). The newly resolved crystal structure of human α1β2γ2 GABA_A_R (PDB ID: 6X3X) with a resolution of 2.92 Å was used as a receptor, which contained a co-crystallized ligand DZP at the BZD binding site [32]. After the pretreatment steps of the original protein, including hydrogenating, adding electric charges, fixing protein side chains and termini chains, removing waters, and extracting the co-crystallized ligand, the applicable binding pocket was generated by a ligand mode. In order to examine the dependability of the docking method, the extracted DZP was firstly redocked into the generated binding pocket by the Surflex-Dock Geom module to inspect whether the redocked ligand conformation and the originally crystallographic conformation overlap. Meanwhile, the conformation differences between the redocked and original ligands were estimated by the RMSD value. An RMSD < 2.0 Å was regarded as a reference standard, suggesting that the used docking method is credible [41]. After that, all selected imidazo [1,2-a]-pyridine PAMs were docked into the active pocket using the same method.

### 3.5. Pharmacophore Model

A pharmacophore is a molecular interaction characteristic shared by a group of active molecules. In this study, ten imidazo [1,2-a]-pyridine as GABA_A_R PAMs (Table 1) with relatively high biological activities and diverse structures were selected to establish pharmacophore models by the genetic algorithm with linear assignment of hyper-molecular alignment of datasets (GALAHAD) module of SYBYL-X 2.1 [46]. The remaining compounds were used for the evaluation of the constructed models. Twenty models were generated, and the model with high values of SPECIFICITY, N_HITS, HBOND, MOL_QRY and the low value of ENERGY was selected for further study. To confirm whether the chosen model was sufficient to recognize the active compounds from the database, it was necessary to evaluate the model using the decoy set method [47]. The potential pharmacophore models were performed to retrieve a decoy set database, which was composed of 3892 inactive compounds (downloaded from http://dud.docking.org/r2/, accessed on 1 October 2020) and 23 active compounds (Table 1) in addition to those used to generate this pharmacophore model. The following two main indexes were used to assess the model: the enrichment factor (*EF*) and the Güner–Henry (*GH*) score, defined by Equations (1) and (2), respectively.

(1) $$EF=\frac{H_{a}\;/\;H_{t}}{A\;/\;D}$$

(2) $$GH=\left[\frac{H_{a}\left(3A+H_{t}\right)}{4H_{t}A}\right]\left.\left(1-\frac{H_{t}-H_{a}}{D-A}\right.\right)$$

*Ha*, *Ht*, *A*, and *D* represent the number of true positive compounds in the database, the number of all hit compounds, the number of true positive compounds in the list, and the total number of compounds in the decoy database, respectively. Generally, a reliable model needs to satisfy *EF* > 1 and 0.6 < *GH* < 1 [36,47].

### 3.6. Virtual Screening

Considering structural diversity and commercial availability, the ZINC purchase database (http://zinc15.docking.org, accessed on 1 November 2020) that contained about 20 million compounds was used for virtual screening in this study. A multi-stage virtual screening was carried out against the database through the combination of the optimal pharmacophore model, the Topomer Search technology, molecular dockings, and ADME/T predictions. In the first stage, the extraction of pharmacophore features from the best pharmacophore model was used as a 3D search query to retrieve potential molecules. The QFIT parameters were used to evaluate the matching degree of screened compounds with the pharmacophore features. Then, the compounds with a QFIT > 50 were selected for the second-round screening of Topomer search. The Topomer Search technology decomposes all database molecules into different groups, and compares topological similarities with the suspicious R groups obtained from the Topomer CoMFA model. Subsequently, the contribution of a series of R groups to the activity was predicted by the established Topomer CoMFA model. In this study, the R_a_ and R_b_ segments were selected as templates (Figure 14). The corresponding R groups were screened from the database, and the TOPDIST and the minimum heavy atom of each fragment were set to 185 and 3, respectively, to evaluate the degree of binding [36]. Target fragments with higher contributions were collected from all screened R groups using compound **14** as a template.

Following, some newly molecules were designed from the combination of the hit R_a_ and R_b_ groups for the next round of docking screening. The hit compounds with a docking score of >9 and good binding patterns were selected for further study. Finally, ADME/T properties of identified hit compounds, compound **14**, and zolpidem were predicted by two web tools of *pkCSM-pharmacokinetics* (http://biosig.unimelb.edu.au/pkcsm/prediction, accessed on 1 November 2020) [37] and *SwissADME* (http://www.swissadme.cn, accessed on 1 November 2020) [38]. Following properties were considered preferentially to obtain the satisfactory compounds: physicochemical properties, lipophilicity, water solubility, blood–brain barrier permeability (logBB), CNS permeability, synthetic accessibility, and toxicity. The hit compounds with desired pharmacophore features and Topomer CoMFA compliance, high docking scores, good binding modes, and ideal ADME/T evaluation results were further studied by MD simulations.

### 3.7. Molecular Dynamics

To further confirm the stability of the hit compounds in the human GABA_A_R, 30 ns MD simulations were performed on different protein complexes using GROMACS 2016.5 software (Uppsala University, Stockholm University, and the Royal Institute of Technology, Sweden). The protein topological parameters were created by the Pdb2gmx module under the AMBER99SB force field [48]. The ligand topological files were automatically generated by the ACPYPE tools. The molecular system was solvated with SPC/E water models with about a 12 Å buffering distance between the protein receptor and the edges of the cubic box, and twenty additional chloride ions for the neutralization of the system. Moreover, the protein–ligand complexes were successively subjected to 100 ps simulations to accomplish the NVT and NPT equilibrium at 300 K and 1 atm [49]. Finally, a 30 ns MD simulation by 2 fs per step was carried out for each complex. A few parameters, such as RMSD, RMSF, Rg and hydrogen bond numbers, were conducted to investigate the stability or variation in all complex systems during the 30 ns dynamic simulation. The binding free energy was calculated by using the MM-PBSA method [39], and the calculation formula of binding free energy was calculated as follows:

(3) $$\Delta G_{bind}=G_{complex}-G_{free-protein}-G_{free-ligand}$$

in which *G_complex_* represents the complex energy, *G_free-protein_* represents the receptor energy and *G_free-ligand_* represents the energy of the unbound ligand. Binding energies were extracted using the MmPbSaStat.py program and key residue contributions towards binding were gained.

## 4. Conclusions

In this study, an integrated set of computational methodologies was used to explore the novel hit compounds of α1-GABA_A_R PAMs. The 3D-QSAR models were constructed based on 33 imidazo [1,2-a]-pyridines, to visually understand the effect of diverse substitutions on the activity of these GABA_A_R PAMs. The molecular docking studies revealed the interaction patterns of these PAMs in the BZD pocket, indicating that these imidazo [1,2-a]-pyridines could form key hydrogen bonds with the residues Ser205 (α1) and Ser206 (α1), and could form hydrophobic or π–π stacking interactions with the residues Phe100 (α1), His102 (α1), Tyr160 (α1), Val203 (α1), Tyr210 (α1), and Phe77 (γ2). These interactions might be essential for the bindings or activities of these GABA_A_R PAMs. The pharmacophore model further confirmed that the hydrophobic aromatic ring and the side chain of the acylamino group were important pharmacophore features. The best pharmacophore model, containing six key features, was in accordance with the 3D-QSAR models and docking results. The virtual screening based on the best pharmacophore model, Topomer Search, molecular dockings, ADME/T predictions, and MD simulations finally confirmed four newly hit compounds (**DS01**–**DS04**). The compounds **DS03** and **DS04** could steadily bind to the 6X3X protein in the dynamic environment, and this was further corroborated by the binding energy analysis; however, its synthesis and biological activity remain to be further studied. We believe that these findings could provide profound theoretical direction and information for the further design and development of novel α1-GABA_A_R PAMs with good antipsychotic activities.

## Figures and Tables

**Table 1:** Chemical structures of the used imidazo [1,2-a]-pyridine derivatives and their actual and predicted p*Ki* values. 
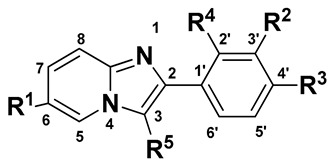

No.	R^1^	R^2^	R^3^	R^4^	R^5^	*Ki*(nM)	Actual p*Ki*	CoMFA		CoMSIA		Topomer CoMFA	
								Pred. p*Ki*	Residual	Pred. p*Ki*	Residual	Pred. p*Ki*	Residual
**1**	CH_3_	H	F	H	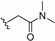	60.0	7.222	7.240	0.018	7.269	0.047	7.190	−0.032
**2 ^a^**	F	H	CH_3_	H	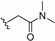	25.0	7.602	7.494	−0.108	7.529	−0.073	7.490	−0.112
**3**	F	H	H	H	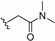	130.0	6.886	6.941	0.055	6.937	0.051	7.020	0.134
**4**	F	F	H	H	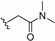	140.0	6.854	6.854	0	6.935	0.081	6.920	0.066
**5 ^a^**	F	H	F	F	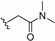	180.0	6.745	6.843	0.098	6.916	0.171	6.920	0.175
**6**	F	H	CF_3_	H	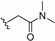	68.0	7.167	7.154	−0.013	7.162	−0.005	7.170	0.003
**7**	CF_3_	H	F	H	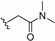	510.0	6.292	6.324	0.032	6.327	0.035	6.330	0.038
**8**	CF_3_	H	F	F	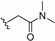	830.0	6.081	6.080	−0.001	6.080	−0.001	6.090	0.009
**9**	CF_3_	H	CF_3_	H	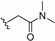	487.0	6.312	6.324	0.012	6.316	0.004	6.320	0.008
**10**	F	H	F	H	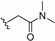	56.0	7.252	7.150	−0.102	7.165	−0.087	7.180	−0.072
**11**	F	F	F	H	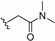	58.0	7.237	7.278	0.041	7.158	−0.079	7.220	−0.017
**12**	CF_3_	F	H	H	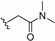	730.0	6.137	6.094	−0.043	6.100	−0.037	6.080	−0.057
**13**	CH_3_	H	F	H	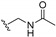	78.4	7.106	7.159	0.053	7.172	0.066	7.230	0.124
**14 ^#^**	CH_3_	H	F	H	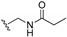	27.2	7.565	7.447	−0.118	7.415	−0.150	7.460	−0.105
**15 ^a^**	CH_3_	H	F	H	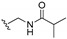	364.0	6.439	6.307	−0.132	6.539	0.100	6.420	−0.019
**16**	CH_3_	H	F	H	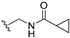	116.0	6.936	6.982	0.046	6.981	0.045	7.030	0.094
**17 ^#^**	F	H	CH_3_	H	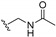	44.0	7.357	7.405	0.048	7.409	0.052	7.380	0.023
**18 ^#^**	F	H	CH_3_	H	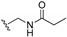	36.5	7.438	7.389	−0.049	7.547	0.109	7.580	0.142
**19 ^a^**	F	H	CH_3_	H	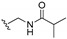	87.5	7.058	7.253	0.195	7.268	0.210	7.310	0.252
**20**	F	H	CH_3_	H	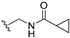	55.1	7.259	7.214	−0.045	7.216	−0.043	7.220	−0.039
**21 ^#^**	F	H	F	H	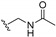	43.0	7.367	7.306	−0.061	7.342	−0.025	7.320	−0.047
**22 ^#^**	F	H	F	H	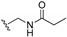	37.0	7.432	7.546	0.114	7.573	0.141	7.480	0.048
**23 ^a^**	F	H	F	H	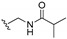	308.7	6.510	6.651	0.141	6.419	−0.091	6.540	0.030
**24 ^a, #^**	F	H	F	H	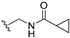	20.9	7.680	7.663	−0.017	7.657	−0.023	7.740	0.060
**25**	F	F	F	H	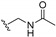	39.0	7.409	7.372	−0.037	7.369	−0.040	7.410	0.001
**26 ^#^**	F	F	F	H	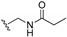	24.0	7.620	7.626	0.006	7.628	0.008	7.560	−0.060
**27**	F	F	F	H	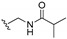	72.0	7.143	7.177	0.034	7.209	0.066	7.170	0.027
**28 ^a,#^**	F	F	F	H	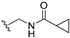	22.8	7.642	7.568	−0.074	7.594	−0.048	7.390	−0.252
**29**	F	H	CF_3_	H	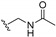	62.7	7.203	7.199	−0.004	7.150	−0.053	7.160	−0.043
**30 ^#^**	F	H	CF_3_	H	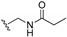	48.0	7.319	7.347	0.028	7.399	0.08	7.260	−0.059
**31**	F	H	CF_3_	H	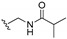	86.3	7.064	7.029	−0.035	6.999	−0.065	6.970	−0.094
**32 ^a^**	F	H	CF_3_	H	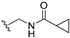	67.0	7.174	7.191	0.017	7.195	0.021	7.070	−0.104
**Zolpidem ^#^**	CH_3_	H	CH_3_	H	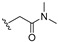	44.0	7.357	7.359	0.002	7.349	−0.008	7.260	−0.097

^a^ The test set molecules used for the 3D-QSAR models. ^#^ The molecules used for the pharmacophore models.

**Table 2:** Internal validation parameters of Topomer CoMFA, CoMFA, and CoMSIA models.

	Model ^a^	q^2^	ONC	SEE	R^2^	F	r_pre_^2^	Field Contribution (%)				
								S	E	H	D	A
Topomer CoMFA	S+E	0.857	7	0.092	0.978	74.312	0.879					
CoMFA	S+E	0.808	15	0.084	0.987	44.347	0.935	0.373	0.627			
CoMSIA	S+E+H+D+A	0.862	13	0.093	0.980	40.610	0.927	0.078	0.180	0.168	0.407	0.167
	S+E+H+D	0.839	13	0.106	0.980	41.865	0.852	0.086	0.215	0.184	0.516	
	S+E+H+A	0.823	10	0.088	0.967	40.690	0.876	0.157	0.339	0.340		0.164
	E+H+D+A	0.870	12	0.091	0.978	46.769	0.926		0.188	0.224	0.411	0.176
	S+E+H	0.815	10	0.109	0.965	38.265	0.839	0.186	0.410	0.404		
	E+H+A	0.839	12	0.099	0.975	39.391	0.892		0.326	0.296		0.378
	S+E+D	0.864	13	0.092	0.980	41.944	0.929	0.202	0.242		0.556	
	E+H+D	0.867	11	0.097	0.974	44.741	0.921		0.250	0.325	0.425	
	S+H	0.817	10	0.105	0.967	41.551	0.823	0.205		0.705		
	E+H	0.820	11	0.111	0.966	33.460	0.845		0.448	0.552		
	H+A	0.805	12	0.094	0.973	44.556	0.814			0.492		0.508

^a^ S, E, H, D, and A mean steric, electrostatic, hydrophobic, hydrogen bond donor, and hydrogen bond acceptor fields, respectively. q^2^: cross-validated correlation coefficient; ONC: optimal number of components; SEE: standard error of estimate; R^2^: non-cross-validated correlation coefficient; F: F-statistic values; r_pred_^2^: predictive correlation coefficient.

**Table 3:** External validation parameters of Topomer CoMFA, CoMFA, and CoMSIA models.

Validation Parameters	RMSE	r^2^	r_0_^2^	r_0_′^2^	(r^2^ − r_0_′^2^)/r^2^	k	k′	r_m_^2^	r_m_′^2^	∆r_m_^2^	$\bar{{r_{m}}^{2}}$
Topomer CoMFA	0.156	0.882	0.881	0.856	0.0296	0.9974	1.0022	0.856	0.739	0.116	0.798
CoMFA	0.114	0.936	0.935	0.927	0.0099	0.9973	1.0019	0.912	0.846	0.065	0.878
CoMSIA(S+E+H+D+A)	0.121	0.944	0.943	0.938	0.0051	0.9863	1.0132	0.942	0.871	0.026	0.884

RMSE: root mean square error for the test set compounds; r^2^: the regression line coefficient of correlation for the test set compounds; r_0_^2^ (predicted vs. observed activities) and r_0_′^2^ (observed vs. predicted activities): the correlation coefficient of regression lines with a zero intercept; k (predicted vs. observed activities) and k′ (observed vs. predicted activities): the slope of regression lines with a zero intercept; r_m_^2^: calculated by [r^2^(1 − (r^2^ − r_0_^2^)^0.5^)]; r_m_′^2^: calculated by [r^2^(1 − (r^2^ − r_0_′^2^)^0.5^)]; Δr_m_^2^ and $\bar{{r_{m}}^{2}}$: the difference and average values between r_m_^2^ and r_m_′^2^.

**Table 4:** Statistical results of the pharmacophore models.

**Name**	**SPECIFICITY**	**N_HITS**	**FEATS**	**PARETO**	**ENERGY**	**STERICS**	**HBOND**	**MOL_QRY**
**MODEL_1**	**5.015**	**10**	**6**	**0**	**25.5**	**421.9**	**35.8**	**33.85**
MODEL_2	5.028	10	6	0	111.93	445.9	35.9	33.1
MODEL_3	5.022	8	6	0	69.9	469.5	33.9	31.02
MODEL_4	5.019	9	6	0	14,028.0703	450.9	36.8	33.57
MODEL_5	5.020	7	6	0	50.97	426.7	32.2	30.02
MODEL_6	5.019	9	6	0	13,991.6396	411.9	36.3	34.27
MODEL_7	5.024	7	6	0	21.15	363	34.7	27.14
MODEL_8	5.025	9	6	0	13.94	366.6	31.1	28.9
MODEL_9	4.255	10	5	0	20.15	396.4	30.4	19.84
MODEL_10	5.023	6	6	0	25.47	416.8	25.8	21.04
MODEL_11	5.018	9	6	0	34.75	353.9	36.5	13.55
MODEL_12	5.026	8	6	0	10.92	328.5	29.8	15.6
MODEL_13	4.982	8	6	1	39.63	413.9	31	26.93
MODEL_14	4.398	10	5	1	46.68	386.8	31.2	26.14
MODEL_15	5.021	9	6	1	55.91	383.1	32.9	25.61
MODEL_16	4.394	10	5	1	36.53	380.1	29.5	27.07
MODEL_17	5.017	8	6	1	39.23	374.4	30.4	24.03
MODEL_18	5.008	7	6	1	55.91	393.9	32.3	17.77
MODEL_19	5.025	7	6	1	14.13	339.4	27.9	25.46
MODEL_20	5.012	9	6	1	65.33	382.1	33.2	16.91

**Table 5:** Predicted ADME/T properties of compounds **DS01–04**, **14**, and zolpidem.

Parameter		Compound					
		DS01	DS02	DS03	DS04	Zolpidem	14
Molecular properties	MW (g/mol)	364.42	390.94	376.91	377.47	307.39	311.36
	logP	2.861	3.853	3.540	3.785	3.248	3.475
	Fraction Csp^3^	0.29	0.37	0.33	0.36	0.26	0.22
	rotatable bonds	5	5	5	8	3	4
	TPSA (Å^2^)	69.09	69.09	69.09	69.09	37.61	46.40
Absorption	Water solubility	−3.076	−3.091	−3.094	−3.357	−3.586	−3.405
	Caco_2_ permeability (log Papp in 10^−6^ cm/s)	1.217	1.008	1.003	1.445	0.977	1.321
	Intestinal absorption (human) (% Absorbed)	94.438	95.073	94.320	96.052	95.252	94.089
	Skin permeability (log Kp)	−2.725	−2.735	−2.735	−2.735	−2.735	−2.735
	GI absorption	High	High	High	High	High	High
	P-gp substrate	No	No	No	No	No	No
Distribution	BBB permeant (log BB)	Yes	Yes	Yes	Yes	Yes	Yes
	CNS permeant (log PS)	−1.054	−1.035	−1.307	−1.295	−1.125	−1.265
Metabolism	CYP2D6 substrate	No	No	No	No	No	No
	CYP3A4 substrate	No	No	No	No	No	No
	CYP2D6 inhibitor	No	No	No	No	No	No
	CYP3A4 inhibitor	No	No	No	No	No	No
Excretion	Total clearance (log mL/min/kg)	0.762	0.842	0.848	0.894	0.722	0.886
	Renal OCT2 substrate	No	No	No	No	Yes	Yes
Toxicity	hERG I inhibitor	No	No	No	No	No	No
	Skin sensitization	No	No	No	No	No	No
Drug-likeness	Lipinski violations	0	0	0	0	0	0
	Synthetic accessibility	3.24	3.46	3.35	3.27	2.93	2.72

MW: Molecular weight; TPSA: the topological polar surface area; GI absorption: gastrointestinal absorption; BBB permeant: blood–brain barrier permeant; CNS permeant: central nervous system permeant; P-gp substrate: P-glycoprotein substrate; renal OCT2 substrate: renal organic cation transporter 2 substrate.

**Table 6:** Chemical structures, docking scores, and predicted p*Ki* values of the newly screened hit compounds.

Compound	Structure	Docking Score	Predicted p*Ki*
**14**	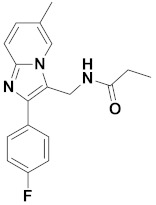	6.910	7.460
**DS01**	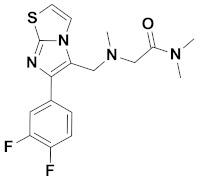	9.035	7.643
**DS02**	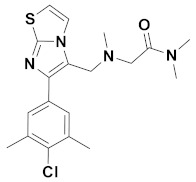	9.036	7.645
**DS03**	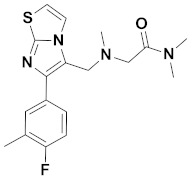	9.776	7.689
**DS04**	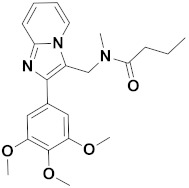	9.262	7.678

**Table 7:** The free binding energies of zolpidem, compound **14**, and the newly screened hit compounds (**DS03** and **DS04**) in the human GABA_A_R (PDB ID: 6X3X).

Complex	ΔE_vdW_ (kJ/mol)	ΔE_ele_ (kJ/mol)	ΔG_PB_ (kJ/mol)	ΔG_SA_ (kJ/mol)	ΔG_binding_ (kJ/mol)
6X3X-**zolpidem**	−166.722 ± 2.866	−12.403 ± 5.571	102.850 ± 2.345	−18.907 ± 0.636	−95.181 ± −6.696
6X3X-**14**	−185.718 ± 5.400	−33.782 ± 4.775	117.687 ± 6.308	−18.241 ± 0.582	−120.055 ± 6.238
6X3X-**DS03**	−187.731 ± 10.951	−14.533 ± 4.779	94.581 ± 4.630	−18.692 ± 0.655	−126.376 ± 11.440
6X3X-**DS04**	−183.268 ± 8.895	−45.446 ± 6.257	116.936 ± 7.296	−19.729 ± 0.823	−131.507 ± 10.246

ΔE_vdW_: van der Waal energy; ΔE_ele_: electrostatic energy; ΔG_PB_: polar solvation energy; ΔG_SA_: SASA energy; ΔG_binding_: binding energy.

## Data Availability

Data is contained within the article and Supplementary Materials.

## Supplementary Materials

The following are available online at https://www.mdpi.com/article/10.3390/ijms22179645/s1.
