# Supplementary material for: In Silico Screening of Novel α1-GABAA Receptor PAMs towards Schizophrenia Based on Combined Modeling Studies of Imidazo [1,2-a]-Pyridines
Source: Int J Mol Sci. 2021 Sep 6;22(17):9645. doi: 10.3390/ijms22179645 (PMC8431797; doi:10.3390/ijms22179645)
Supplement: Supplementary file 1 [file ijms-22-09645-s001.zip › ijms-1326845-supplementary.pdf]

Table S1. Validation parameters of the pharmacophore model using a decoy set method.

number of active compounds in the hit list (Ha)	20
number of all hit compounds (Ht)	29
number of active compounds in the database (A)	23
number of compounds in the database (D)	3915
enrichment factor (EF)<br>[(Ha/Ht)/(A/D)]	117.398
Güner−Henry score (GH)<br>[(Ha(3A + Ht)/4HtA) × (1 − (Ht − Ha)/(D − A))]	0.734

Table S2. The RMSF values of key residues of four complex systems in the 6X3X BZD site.

	6X3X-zolpidem (nm)	6X3X-**14** (nm)	6X3X-**DS03** (nm)	6X3X-**DS04** (nm)
Phe100 (loop A)	0.057	0.067	0.050	0.055
His102 (loop A)	0.054	0.071	0.046	0.051
Tyr160 (loop B)	0.059	0.086	0.055	0.056
Val203 (loop C)	0.064	0.074	0.052	0.057
Ser205 (loop C)	0.068	0.058	0.047	0.046
Ser206 (loop C)	0.091	0.066	0.054	0.042
Tyr210 (loop C)	0.059	0.061	0.048	0.050
Phe77 (loop D)	0.046	0.053	0.040	0.042
Met130(loop E)	0.047	0.065	0.050	0.060
Thr142(loop E)	0.059	0.064	0.056	0.057
